# Higher subcutaneous adipose tissue radiodensity is associated with increased mortality in patients with cirrhosis

**DOI:** 10.1016/j.jhepr.2022.100495

**Published:** 2022-04-27

**Authors:** Maryam Ebadi, Abha R. Dunichand-Hoedl, Elora Rider, Norman M. Kneteman, James Shapiro, David Bigam, Khaled Dajani, Vera C. Mazurak, Vickie E. Baracos, Aldo J. Montano-Loza

**Affiliations:** 1 Division of Gastroenterology and Liver Unit, University of Alberta Hospital, Edmonton, Alberta, Canada; 2 Division of Human Nutrition, University of Alberta, Edmonton, Alberta, Canada; 3 Division of Transplantation, Department of Surgery, University of Alberta Hospital, Edmonton, Alberta, Canada; 4 Department of Oncology, Cross Cancer Institute, Edmonton, Alberta, Canada

**Keywords:** Computed tomography, CT attenuation, Outcomes, End-stage liver disease, CT, computed tomography, HCC, hepatocellular carcinoma, HU, Hounsfield units, L3, third lumbar vertebra, LT, liver transplant, NASH, non-alcoholic steatohepatitis, SAT, subcutaneous adipose tissue, sHR, sub-distribution hazard ratio

## Abstract

**Background & Aims:**

Association between sarcopenia and mortality in cirrhosis is well recognised; however, little is known about the clinical implications of adipose tissue radiodensity, indicative of biological features. This study aimed to determine an association between high subcutaneous adipose tissue (SAT) radiodensity and survival, compare the prevalence of high SAT radiodensity between healthy population and patients with cirrhosis, and identify an association between computed tomography (CT)-measured SAT radiodensity and histological characteristics.

**Methods:**

Adult patients with cirrhosis (n = 786) and healthy donors (n = 129) with CT images taken as part of the liver transplant (LT) assessment were included. Abdominal SAT biopsies (1–2 g) were harvested from the incision site at the time of LT from 12 patients with cirrhosis.

**Results:**

The majority of patients were male (67%) with a mean model for end-stage liver disease (MELD) score of 15 ± 8. SAT radiodensity above -83 HU in females (sub-distribution hazard ratio [sHR] 1.84, 95% CI 1.20–2.85, *p* = 0.006) and higher than -74 HU in males (sHR 1.51, 95% CI 1.05–1.18, *p* = 0.02) was associated with the highest mortality risk after adjusting for confounders in competing risk analysis. The frequency of high SAT radiodensity was 26% for those with cirrhosis, compared with 2% in healthy donors (*p* <0.001). An inverse correlation was found between SAT radiodensity and the mean cross-sectional area of SAT adipocytes (*r* = -0.67, *p* = 0.02). Shrunken, smaller adipocytes with expanded interstitial space were predominant in patients with high SAT radiodensity, whereas larger adipocytes with a thin rim of cytoplasm were observed in patients with low SAT radiodensity (744 ± 400 *vs.* 1,521 ± 1,035 μm^2^, *p* <0.001).

**Conclusion:**

High SAT radiodensity frequently presents and is associated with a higher mortality in cirrhosis. SAT morphological rearrangement in patients with high SAT radiodensity might indicate diminished lipid stores and alterations in tissue characteristics.

**Lay summary:**

Poor quality of subcutaneous adipose tissue (fat under the skin) is associated with higher mortality in patients with end-stage liver disease. Fat cells are smaller in patients with poor adipose tissue quality.

## Highlights

• Subcutaneous adipose tissue (SAT) radiodensity can be objectively measured by CT in Hounsfield units (HU).
• SAT radiodensity constitutes an indirect surrogate marker of adipose tissue quality.
• High SAT radiodensity in cirrhosis was defined as >-83 HU in females and >-74 HU in males.
• High SAT radiodensity presents in more than a quarter of patients with cirrhosis and is associated with higher mortality.
• High SAT radiodensity might indicate adipose tissue remodelling with morphological features of atrophy.

## Graphical abstract

**Figure:**
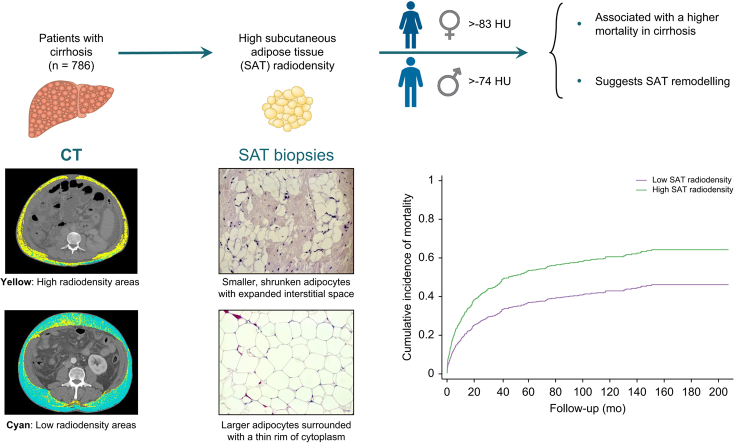

## Introduction

Over the past decade, there has been increasing recognition of the relevance of body composition evaluation in patients with cirrhosis.^1^ Image-based modalities such as computed tomography (CT) scans have extended our current knowledge on the prognostic significance of body composition parameters in patients with cirrhosis. Body composition analysis at the time of liver transplant (LT) evaluation indicated a relationship between abnormal body composition features and poor prognosis in cirrhosis. Sarcopenia (low muscle mass) is a common complication in patients with cirrhosis that has been extensively investigated in this patient population.^2^ Although the independent association between this radiologically identified abnormality in skeletal muscle and poor prognosis in cirrhosis is well recognised,^1^ little is known about the clinical implications of adipose tissue radiodensity, which reflects tissue biological features. The concept of adipose tissue radiodensity is emerging in literature, and its relationship to prognosis has been explored within the oncology setting.^3^^,^^4^

Adipose tissue radiodensity can be objectively measured by CT in Hounsfield units (HU). CT-derived average adipose tissue radiodensity has been introduced as an indirect surrogate marker of adipose tissue quality.^5^ Several potential factors, such as blood flow,^6^ adipocyte size,^7^ lipid content,^8^ and fluid-to-triglyceride ratio^9^ might impact radiodensity measured by CT HU. Therefore, HU is not a sole marker of stored triglycerides in adipose tissue; it also represents other aspects of tissue structure including water, blood, and residual fat cell components.^9^ However, histological data on adipose tissue quality features are inadequate because of the invasiveness of tissue sampling and therefore, indirect, accessible surrogate markers are required.

Lower adipose tissue radiodensity (*i.e.* more negative CT radiodensity) has been linked to the larger lipid droplets within hypertrophic adipocytes.^8^ A recent study revealed that adipose tissue radiodensity is a more robust predictor of insulin resistance than adipose tissue volume in obese and overweight individuals.^10^ Subcutaneous adipose tissue (SAT), provides energy in hypercatabolic states such as cirrhosis and stimulates insulin response, glucose and lipid metabolism, as well as the immune response by secreting adipokines, primarily leptin.^11^^,^^12^ Higher SAT radiodensity might represent adipose tissue fibrosis.^10^ Having higher SAT radiodensity was associated with shorter survival in patients with multiple myeloma.^3^ In addition, increased SAT radiodensity has also been linked to the risk of decompensation in patients with cirrhosis.^13^

Given the potential prognostic importance of high SAT radiodensity, the primary objective of this study was to determine whether there is an association between higher SAT radiodensity and survival, independent of sarcopenia, in patients with cirrhosis. Secondly, SAT radiodensity and the prevalence of high SAT radiodensity was compared between patients with cirrhosis and healthy controls. Lastly, an association between CT-measured SAT radiodensity and histological characteristics was determined.

## Patients and methods

### Study population

This study was reviewed and approved by the Institutional Review Board of the University of Alberta (Pro00066572). Adult patients with cirrhosis (n = 786) and healthy controls (n = 129) who had a CT image acquired as part of the routine LT assessment at the University of Alberta Hospital between August 1996 and October 2019 were included. Patients’ clinical and demographic features were collected from the Alberta Liver Transplant database. Adult donors for living LT between the age of 19 and 58 who tested negative for viral hepatitis B and C serologies, without prior hepatic resections, without major abdominal surgeries, without a history of alcohol abuse and illicit drug use, and with no evidence of non-alcoholic fatty liver disease served as healthy controls.

### CT image analysis

Subcutaneous adipose tissue cross-sectional area and radiodensity were measured using analysis of abdominal CT scans taken at the third lumbar vertebra (L3), as part of the LT assessment. L3 has been selected as a consistent landmark, as adipose tissue areas taken at this level from a single CT image have the best correlation with total body adipose tissue mass.^14^ The cross-sectional area for SAT and skeletal muscle were calculated on an axial single image at L3 using standard HU thresholds of -190 to -30 HU^15^ for SAT and of -29 to 150 HU for skeletal muscle^16^ using Slice-O-Matic software (V4.2; Tomovision, Montreal, QC, Canada; Supplementary CTAT Table). Applying these HUs, adipose tissue between the skin line and outer abdominal wall was quantified as SAT. The sum of SAT and skeletal muscle cross-sectional areas (cm^2^) were divided by the square of the height in meters (m^2^) and reported as SAT index and skeletal muscle index (cm^2^/m^2^), respectively. Sarcopenia was defined using established cut-offs in patients with cirrhosis awaiting LT as skeletal muscle index <39 cm^2^/m^2^ in females and <50 cm^2^/m^2^ in males.^17^ SAT radiodensity was reported as the mean value for the entire SAT area.

### LT recipients and subcutaneous adipose tissue biopsies

Adult patients with cirrhosis listed for LT at the University of Alberta LT centre were consecutively approached between November 2017 and August 2018 for SAT biopsy collection during LT procedure. Of the 20 patients who consented, we were able to collect biopsies from 12 patients. Biopsies from lower abdominal SAT (1–2 g) were harvested from the incision site at the start of LT surgery using sharp dissection and without the use of electrocautery. Abdominal CT images of these 12 patients were analysed to determine an association between radiodensity and histological characteristics of SAT.

### Adipose tissue morphometry

SAT samples were fixed in 10% paraformaldehyde for 24 h, dehydrated in absolute ethanol, and cleared in xylene. Paraffin-embedded tissue sections were then cut into 5 μm sections, stained with Harris haematoxylin, and counterstained with eosin. Images were acquired with light microscopy (Olympus Qlmaging micropublisher camera) and visualised at 20× magnification. Adipocyte size was determined by measuring cross-sectional areas of cells (>300 cells counted/group) in 4 random fields using Image J software (National Institutes of Health; http://rsb.info.nih.gov/ij/). A haemacytometer was used as a calibrator for measuring the size of adipocytes.

### Statistical analysis

Continuous variables are reported as mean and SD, and differences in means were compared using an independent *t* test. Descriptive statistics for categorical variables are presented as frequency, and the comparison between study groups was performed using the Pearson χ^2^ test. Correlation between SAT index and radiodensity was determined by Pearson’s correlation coefficient (*r*) analysis in 786 patients with cirrhosis.

The primary outcome of interest in this study was mortality, defined as death before LT or de-listing for clinical deterioration. However, in these patients, death and LT are 2 competing events, and therefore, competing risk analysis was conducted using the Fine–Gray sub-distribution hazard model to estimate predictors of mortality in the presence of competing events. A competing risk analysis is a more robust approach, compared with the conventional survival analysis, in the presence of competing events.^18^ Significant predictors of mortality were determined using univariate and multivariate Fine–Gray sub-distribution hazard models, and the results were reported as sub-distribution hazard ratios (sHRs) with 95% CI. Features known to be associated with mortality of patients with cirrhosis, including age, cirrhosis aetiology, serum albumin and sodium, model for end-stage liver disease (MELD) score, refractory ascites, hepatic encephalopathy, and variceal bleeding were included in univariate analysis.^19^^,^^20^

The impact of sex on the biological role, distribution, and characteristics of adipose tissue has been extensively reviewed,[21], [22], [23], [24] suggesting the necessity to establish sex-specific cut-offs. SAT radiodensity cut-offs to predict mortality by sex were established using a receiver-operating characteristic analysis. The value with the highest Youden’s index (sensitivity + specificity − 1) was considered as the optimal cut-off. Variables with *p* <0.10 in the univariate analysis were included in the multivariate model. Competing risk analysis to estimate the cumulative incidence of mortality was used, according to the SAT radiodensity cut-off. Survival over time was calculated using methods of Kaplan–Meier, and curves were compared using the log-rank (Mantel–Cox) test.

In the 12 patients who underwent LT, non-parametric data were analysed using the Wilcoxon–Mann–Whitney test. Spearman’s rank correlation test was used to determine the relationship between SAT radiodensity and adipocyte size in LT recipients. Statistical analyses were conducted using Stata 15.0 and SPSS 26.0 (Supplementary CTAT Table) and a *p* value <0.05 was considered as a statistically significant difference.

## Results

### Baseline patients characteristics

Patients’ clinical characteristics at the time of CT are shown in Table 1. The majority were male (67%) with a mean age of 56 ± 8 years and a MELD score of 15 ± 8. Hepatitis C (41%), alcohol (24%), non-alcoholic steatohepatitis (NASH; 19%), and hepatitis B (7%) were the main reasons for cirrhosis. Concomitant hepatocellular carcinoma (HCC) was present in 43% of patients evaluated for LT. Evaluated patients were followed up for a mean time of 24 ± 35 months, until death (n = 336), LT (n = 329), or censoring (n = 121).

**Table 1:** **Clinical features associated with mortality at the time of body composition assessment in univariable competing risk analyses**.

Characteristics	All patients (n = 786)	sHR (95% CI)	*p* value
Age (years)	56 ± 8	0.99 (0.98–1.004)	0.23
Sex, female	262 (33)	0.99 (0.79–1.24)	0.94
Cirrhosis aetiology			
Alcohol	189 (24)	1.08 (0.84–1.37)	0.56
Hepatitis C	324 (41)	0.93 (0.75–1.15)	0.49
Hepatitis B	55 (7)	1.02 (0.65–1.60)	0.92
NASH	152 (19)	1.24 (0.96–1.60)	0.09
ALD	62 (8)	0.57 (0.37–0.90)	0.02
Encephalopathy	285 (36)	1.82 (1.42–2.34)	<0.001
HCC	338 (43)	1.16 (0.94–1.43)	0.17
Refractory ascites	207 (26)	1.63 (1.24–2.16)	0.001
Variceal bleeding	140 (18)	2.25 (1.58–3.22)	<0.001
Albumin (g/L, 35–50)	32 ± 6	0.99 (0.97–1.01)	0.18
Sodium (mmol/L, 133–146)	136 ± 6	0.999 (0.98–1.02)	0.95
MELD score	15 ± 8	1.02 (1.01–1.04)	0.002
BMI (kg/m^2^)	27 ± 6	1.01 (0.99–1.03)	0.42
SAT radiodensity (HU)	-87 ± 16	1.01 (1.00–1.01)	0.04
SAT cross-sectional area (cm^2^)	162 ± 112	1.00(0.998–1.002)	0.15
SAT index (cm^2^/m^2^)	60 ± 40	1.00 (0.998–1.003)	0.64
Visceral adipose tissue radiodensity (HU)	-78 ± 10	0.999 (0.99–1.01)	0.90
Sarcopenia∗	277 (35)	1.39 (1.12–1.74)	0.003

Numbers in parentheses are percentages. sHRs and *p* values were estimated using the Fine–Gray sub-distribution hazard model.

ALD, autoimmune liver diseases; HCC, hepatocellular carcinoma; HU, Hounsfield units; MELD, model for end-stage liver disease, NASH, non-alcoholic steatohepatitis; SAT, subcutaneous adipose tissue; sHR, sub-distribution hazard ratio; SMI, skeletal muscle index.

∗ Sarcopenia was defined using established cut-offs in patients with cirrhosis as SMI <50 cm^2^/m^2^ in males and <39 cm^2^/m^2^ in females.^17^

### SAT radiodensity and mortality

Using competitive risk analysis, cirrhosis aetiology, MELD score, refractory ascites, variceal bleeding, hepatic encephalopathy, sarcopenia, and SAT radiodensity (sHR 1.01, 95% CI 1.00–1.01, *p* = 0.04) were predictors of mortality in univariate analysis (Table 1). SAT radiodensity above -83 HU in females (sHR 1.87, 95% CI 1.29–2.70, *p* = 0.001) and higher than -74 HU in males (sHR 1.58, 95% CI 1.13–2.21, *p* = 0.007) were found to be statistically significant values that provide satisfactory discrimination of mortality risk between patients. Compared with female patients with low SAT radiodensity (**≤**-83), high SAT radiodensity (>-83 HU) was associated with the highest mortality risk after adjusting for other confounding factors (sHR 1.84, 95% CI 1.20–2.85, *p* = 0.006; Table 2). Male patients with SAT radiodensity >-74 HU had a higher risk of mortality (sHR 1.51, 95% CI 1.05–1.18, *p* = 0.03; Table 2) compared with the patients with low SAT radiodensity.

**Table 2:** **Clinical parameters associated with mortality in a competing risk model, stratified by sex**.

Characteristics	Univariate		Multivariate	
	sHR (95% CI)	*p* value	sHR (95% CI)	*p* value
**Female patients**				
Age (years)	0.99 (0.97–1.004)	0.13	1.00 (0.98–1.02)	0.72
Cirrhosis aetiology				
Alcohol	1.31 (0.84–2.05)	0.23		
Hepatitis C	0.76 (0.50–1.16)	0.20		
Hepatitis B	1.78 (0.83–3.82)	0.14		
NASH	1.38 (0.93–2.06)	0.11		
ALD	0.58 (0.35–0.96)	0.03	0.47 (0.27–0.80)	0.005
Albumin (g/L)	0.96 (0.92–0.99)	0.01	0.98 (0.94–1.01)	0.18
MELD score	1.03 (1.01–1.06)	0.008	1.05 (1.02–1.09)	0.003
Refractory ascites	2.55 (1.44–4.51)	0.001	2.48 (1.29–4.78)	0.007
Sodium (mmol/L)	0.99 (0.96–1.03)	0.67		
Encephalopathy	2.42 (1.51–3.88)	<0.001	2.29 (1.31–3.98)	0.003
Variceal bleeding	3.93 (1.67–9.25)	0.002	2.85 (1.11–7.37)	0.03
BMI (kg/m^2^)	0.99 (0.96–1.02)	0.57		
SAT cross-sectional area (cm^2^)	1.00 (0.99–1.002)			
SAT index (cm^2^/m^2^)	1.00 (0.99–1.003)	0.75		
HCC	1.18 (0.80–1.74)	0.41		
Sarcopenia∗	1.39 (0.92–2.10)	0.12		
High SAT radiodensity (>-83 HU)	1.87 (1.29–2.70)	0.001	1.84 (1.20–2.85)	0.006
**Male patients**				
Age (years)	1.00 (0.98–1.01)	0.74	1.00 (0.99–1.02)	0.79
Cirrhosis aetiology				
Alcohol	0.99 (0.74–1.34)	0.97		
Hepatitis C	1.00 (0.77–1.30)	0.99		
Hepatitis B	0.89 (0.52–1.51)	0.66		
NASH	1.16 (0.83–1.61)	0.39		
ALD	0.36 (0.09–1.38)	0.14		
Albumin (g/L)	1.00 (0.98–1.02)	0.94		
MELD score	1.02 (0.999–1.04)	0.06	1.04 (1.01–1.06)	0.002
Refractory ascites	1.38 (1.00–1.90)	0.05	1.26 (0.85–1.87)	0.24
Sodium (mmol/L)	1.00 (0.98–1.02)	0.85		
Encephalopathy	1.61 (1.20–2.17)	0.001	1.72 (1.20–2.47)	0.003
Variceal bleeding	1.90 (1.28–2.82)	0.001	1.79 (1.13–2.84)	0.01
BMI (kg/m^2^)	1.02 (0.99–1.04)	0.13		
SAT cross-sectional area (cm^2^)	1.00 (0.998–1.001)	0.98		
SAT index (cm^2^/m^2^)	1.00 (0.997–1.01)	0.38		
HCC	1.17 (0.90–1.52)	0.24		
Sarcopenia∗	1.41 (1.08–1.83)	0.01	1.53 (1.15–2.03)	0.004
High SAT radiodensity (>-74 HU)	1.58 (1.13–2.21)	0.007	1.51 (1.05–1.18)	0.03

sHRs and *p* values were estimated using the Fine–Gray sub-distribution hazard model.

ALD, autoimmune liver diseases; HCC, hepatocellular carcinoma; HU, Hounsfield units; MELD, model for end-stage liver disease; NASH, non-alcoholic steatohepatitis; SAT, subcutaneous adipose tissue; sHR, sub-distribution hazard ratio; SMI, skeletal muscle index.

∗ Sarcopenia was defined using established cut-offs in patients with cirrhosis as SMI <50 cm^2^/m^2^ in males and <39 cm^2^/m^2^ in females.^17^

There was a weak linear correlation between sarcopenia and SAT radiodensity (*r* = 0.20, *p* <0.001). In a subgroup analysis excluding patients with sarcopenia, high SAT radiodensity remained associated with mortality in both females (sHR 1.79, 95% CI 1.06–3.04, *p* = 0.03) and males (sHR 1.79, 95% CI 1.02–3.13, *p* = 0.04; Table S1), suggesting that the prognostic significance of high SAT radiodensity in predicting mortality is independent of sarcopenia.

**Fig. 1:**
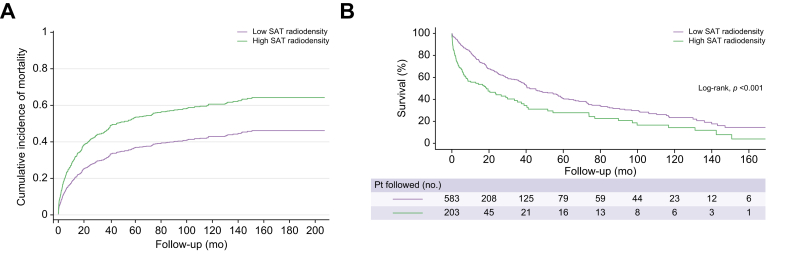
Cumulative incidence (Fine and Gray) of mortality and Kaplan–Meier survival curves among patients with high and low SAT radiodensity. (A) The cumulative incidence functions for patients with high SAT radiodensity and those with low SAT radiodensity were plotted and compared using the sub-distribution hazard as proposed by Fine and Gray. Patients with high SAT radiodensity had a higher cumulative incidence for mortality. (B) Kaplan–Meier curves were applied to estimate survival over time, and the comparison between curves was performed using the log-rank test. Shorter median survival was noticed in patients with high SAT radiodensity than in patients with low SAT radiodensity (log-rank test, *p* <0.001). SAT, subcutaneous adipose tissue.

Cumulative incidence functions for mortality were performed using the parameter estimates of the Fine and Gray model, considering the high SAT radiodensity cut-offs (>-83 in females and >-74 in males). The cumulative incidence of mortality was higher in patients with high SAT radiodensity (Fig. 1A).

Median survival for patients with high SAT radiodensity was 19 months (95% CI 9–29), compared with 42 months (95% CI 33–51) in patients with low SAT radiodensity (*p* <0.001, log-rank test; Fig. 1B). One-year probability of survival was 56 and 79%, for patients with high SAT radiodensity and those with low SAT radiodensity, respectively, and the 5-year probability of survival was 28 and 41% in these same groups.

### Characteristics of patients with high SAT radiodensity

**Fig. 2:**
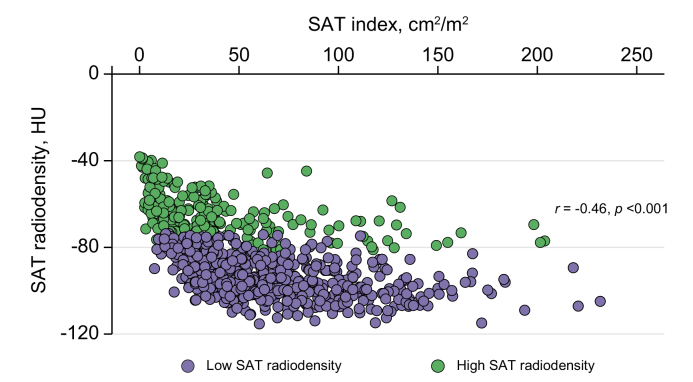
Scatter graph depicting correlations between subcutaneous adipose tissue index and radiodensity. Weak correlation (Pearson’s correlation) between SAT index and radiodensity in patients with cirrhosis (*r* = -0.46, *p* <0.001). HU, Hounsfield units; SAT, subcutaneous adipose tissue.

A significant linear (*p* <0.001) but weak (*r* = -0.46) correlation was observed between SAT radiodensity and SAT index (Fig. 2). High SAT radiodensity was more common in females. HCV-cirrhosis and HCC were less common in patients with high SAT radiodensity who were younger. BMI, SAT index, and serum albumin and sodium levels were lower in patients with high SAT radiodensity, whereas the MELD score was higher (Table 3). The frequency of refractory ascites, hepatic encephalopathy, and sarcopenia was also higher in these patients. No significant difference was observed between patients with low SAT radiodensity and those with high SAT radiodensity regarding alcohol or NASH-related cirrhosis or the frequency of variceal bleeding (Table 3).

**Table 3:** **Clinical features associated with high SAT radiodensity**.

Characteristics	High SAT radiodensity (n = 203)	Low SAT radiodensity (n = 583)	*p* value
Age (years)	54 ± 9	57 ± 8	<0.001
Sex, female	102 (50)	160 (27)	<0.001
Age and sex			
Young male	13 (6)	23 (4)	0.17
Old male	88 (44)	396 (68)	<0.001
Young female	21 (55)	17 (45)	<0.001
Old female	80 (40)	143 (25)	<0.001
Cirrhosis aetiology			
Alcohol	55 (27)	134 (23)	0.25
Hepatitis C	60 (30)	264 (45)	<0.001
Hepatitis B	16 (8)	39 (7)	0.63
NASH	43 (21)	109 (19)	0.47
ALD	28 (14)	34 (6)	<0.001
Albumin (g/L)	30 ± 7	33 ± 6	<0.001
MELD score	19 ± 8	13 ± 7	<0.001
Refractory ascites	71 (35)	136 (23)	0.002
Sodium (mmol/L)	134 ± 6	136 ± 6	<0.001
Encephalopathy	93 (46)	192 (33)	0.001
Variceal bleeding	39 (19)	101 (17)	0.59
HCC	51 (25)	287 (49)	<0.001
BMI (kg/m^2^)	26 ± 7	28 ± 5	
SAT cross-sectional area (cm^2^)	99 ± 90	187 ± 111	<0.001
SAT index (cm^2^/m^2^)	42 ± 40	66 ± 38	<0.001
SAT radiodensity∗ (HU)	-65 ± 11	-94 ± 9	<0.001
Sarcopenia†	93 (46)	184 (32)	<0.001

Independent *t* test for continuous variables and Pearson χ^2^ test for categorical variables were used. HCC, hepatocellular carcinoma; HU, Hounsfield units; MELD, model for end-stage liver disease; NASH, non-alcoholic steatohepatitis; SAT, subcutaneous adipose tissue; SMI, skeletal muscle index.

∗ High SAT radiodensity was defined as SAT radiodensity >-83 HU in females and >-74 HU in males.

† Sarcopenia was defined using established cut-offs in patients with cirrhosis as SMI <50 cm^2^/m^2^ in males and <39 cm^2^/m^2^ in females.^17^

### Adipose tissue radiodensity in donors and patients with cirrhosis

Adipose tissue radiodensity was compared between healthy donors and patients with cirrhosis. SAT radiodensity was significantly lower in donors than in patients with cirrhosis (-101 ± 8 *vs.* -87 ± 16 HU, *p* <0.001). The frequency of high SAT radiodensity was 26% for patients with cirrhosis compared with 2% in donors (*p* <0.001; Table S2).

### Adipose tissue histological characteristics

Of the 12 patients who provided biopsies from lower abdominal SAT, 42% were female with a mean age of 48 ± 11 years and an MELD score of 21 ± 7 points at the time of LT. High SAT radiodensity was present in 33% of patients, and no significant difference in age at LT, sex, MELD score, serum sodium and albumin, sarcopenia presence, and HCC was observed between patients with high SAT radiodensity and those with low SAT radiodensity (Table 4). BMI (22 ± 1 *vs.* 26 ± 4 HU, *p* = 0.03), SAT index (28 ± 18 *vs.* 74 ± 36 HU, *p* = 0.01), SAT radiodensity (-63 ± 11 *vs.* -92 ± 8 HU, *p* = 0.004), and mean SAT adipocyte size (744 ± 400 *vs.* 1,521 ± 1,035 μm^2^, *p* <0.001) were significantly lower in patients with high SAT radiodensity than in those with low SAT radiodensity.

**Table 4:** **Clinical characteristics of 12 liver transplant recipients who had biopsies from lower abdominal SAT**.

Characteristics	High SAT radiodensity (n = 4)	Low SAT radiodensity (n = 8)	*p* value
Age at liver transplant	42 ± 8	51 ± 12	0.28
Sex, female	3 (75)	2 (25)	0.15
MELD score	24 ± 5	19 ± 8	0.11
Sodium (mmol/L)	133 ± 3	135 ± 3	0.28
Albumin (g/L)	30 ± 5	33 ± 9	0.57
HCC	0 (0)	2 (25)	0.42
BMI, kg/m^2^	22 ± 1	26 ± 4	0.03
SAT radiodensity∗ (HU)	-63 ± 11	-92 ± 8	0.004
SAT cross-sectional area (cm^2^)	76 ± 47	221 ± 108	0.005
SAT index (cm^2^/m^2^)	28 ± 18	74 ± 36	0.01
Subcutaneous adipocyte cross-sectional area (μm^2^)	744 ± 400	1,521 ± 1,035	<0.001
Sarcopenia†	2 (50)	3 (38)	0.68

Non-parametric tests were used. HCC, hepatocellular carcinoma; HU, Hounsfield units; MELD, model for end-stage liver disease; SAT, subcutaneous adipose tissue; SMI, skeletal muscle index.

∗ High SAT radiodensity was defined as SAT radiodensity >-83 HU in females and >-74 HU in males.

† Sarcopenia was defined using established cut-offs in patients with cirrhosis as SMI <50 cm^2^/m^2^ in males and <39 cm^2^/m^2^ in females.^17^

**Fig. 3:**
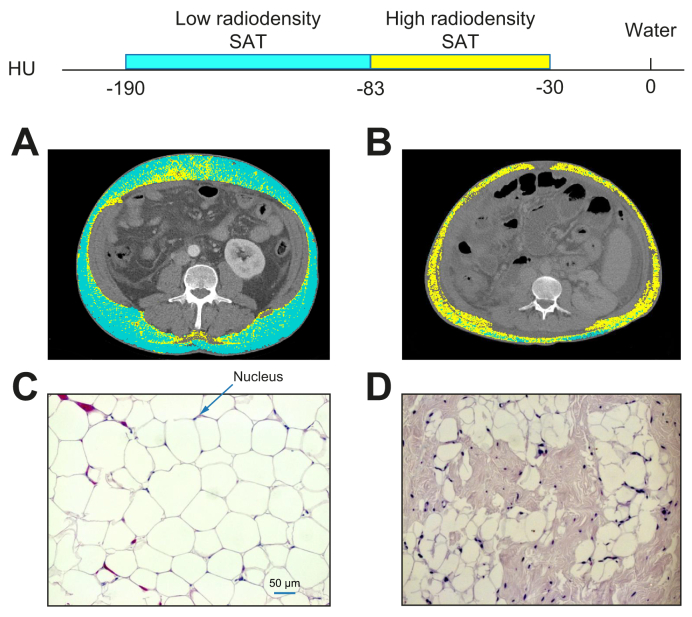
Abdominal CT images taken at the third lumbar vertebra and SAT morphological characteristics of 2 patients with cirrhosis applied for SAT radiodensity assessment. Comparison of 2 patients (A) with low SAT radiodensity (-95 HU) and (B) with high SAT radiodensity of -58 HU. High SAT radiodensity (-30 to -82 HU) is shown in yellow, and low SAT radiodensity (-83 to -190 HU) is shown in cyan. Example images of corresponding to SAT adipocytes stained with H&E (magnification 20×) from patients with high SAT radiodensity and those with low radiodensity. (C) Larger adipocytes with higher cell cross-sectional area (2,122 ± 1,100 μm^2^), surrounded with a thin rim of cytoplasm whose nuclei are compressed to the peripheral rim were observed in a patient with low SAT radiodensity. (D) Shrunken, smaller, polygonal-shape adipocytes (768 ± 443 μm^2^) with distinct alterations in the extracellular matrix, by contrast, were predominant in a patient with high SAT radiodensity. Bar = 50 μm. CT, computed tomography; HU, Hounsfield units; SAT, subcutaneous adipose tissue.

A significant inverse correlation was found between SAT radiodensity and the mean cross-sectional area of adipocytes within the SAT depot (*r* = -0.67, *p* = 0.02). In agreement with this, Fig. 3 highlights the SAT radiodensity estimation at L3 from 2 patients in the high and low SAT radiodensity groups who underwent LT and their corresponding morphological analysis, which is representative of the features observed in each group. Fig. 3A presents a patient with a mean low SAT radiodensity (-95 HU), whereas Fig. 3B presents a patient who had a high SAT radiodensity (-58 HU). Increased mean SAT attenuation is presented as an increase in the areas of high-radiodensity SAT (-30 to -82 HU). Areas demarked in cyan as low radiodensity (-83 to -190 HU) were predominant in Fig. 3A, whereas most of the total SAT areas in Fig. 3B were areas composed of a high radiodensity SAT (-30 to -82 HU), tinted in yellow. This increase in SAT radiodensity was detectable at the microscopic level. Examples of adipocytes stained with H&E from the high and low SAT radiodensity groups are presented in Fig. 3C and D. Morphological analysis showed modifications in SAT histological characteristics with differences in the shape of adipocytes and expanded interstitial space by increasing SAT radiodensity. Larger adipocytes (2,122 ± 1,100 μm^2^) with higher cell cross-sectional area, arranged in close contact with each other and surrounded with a thin rim of cytoplasm whose nuclei are compressed to the peripheral rim, were observed in a patient with low SAT radiodensity (Fig. 3C). Shrunken, smaller, polygonal-shape adipocytes (768 ± 443 μm^2^), by contrast, were predominant in a patient with high SAT radiodensity (Fig. 3D). Distinct alterations in the extracellular matrix with expanded interstitial space and infiltrated mononuclear cells were also observed, indicating cellular function impairment.

## Discussion

The prognostic significance of SAT radiodensity in predicting mortality in patients with cirrhosis was investigated in this study. High SAT radiodensity was frequent in patients with cirrhosis compared with healthy donors and reveals a novel indicator of mortality risk in these patients. Patients with cirrhosis and high SAT radiodensity had a higher frequency of complications than those with low radiodensity. Similar findings have been reported for the association between high SAT radiodensity and risk of decompensation in patients with cirrhosis,^13^ as well as survival in patients with multiple myeloma.^3^ Therefore, this study proposes a novel objective approach to estimate the risk of mortality in patients with cirrhosis, given the radiologically identified abnormalities in adipose tissue.

Association between high SAT radiodensity and mortality in patients with cirrhosis might be reflective of diminished lipid storage capacity of SAT. Subcutaneous adipose tissue plays a central role in lipid storage and energy homoeostasis; high SAT radiodensity might mirror the severe energy exhaustion triggered by cirrhosis, resulting in unfavourable clinical outcomes. However, adipose tissue has been recently appreciated for its comprehensive endocrine function with its adipokines involved in regulating various metabolic and inflammatory states.^11^ In this regard, adipose tissue cellular composition (type of adipocytes, macrophages, etc.) plays an important role in regulating its response to metabolic state, the released adipokines, and its potential impact on other tissues. Therefore, high SAT radiodensity not only is a representative of a lower amount of SAT but also suggests adipose tissue remodelling with morphological features of atrophy demonstrated by smaller, shrunken adipocytes with expanded interstitial space and infiltrated mononuclear cells. Macrophages surrounding damaged or necrotic adipocytes can form crown-like structures and therefore contribute to the inflammatory-fibrotic process.^25^ It has been shown in cancer cachexia that adipose tissue remodelling occurs at the early stages of cachexia; it is linked to adipocyte atrophy, inflammation, and adipose tissue browning, which might eventually lead to adipose tissue dysfunction.^26^ Adipocyte dysfunction impacts its ability to produce fatty acids and adipokines, resulting in whole-body metabolic dysfunction. Thus, high SAT radiodensity may be an indicator of alterations in adipose tissue mass, function, and structure. A lower amount of subcutaneous fat (SAT index <60 cm^2^/m^2^) has been recently identified as an independent predictor of higher mortality in female patients with cirrhosis.^27^ Of 96 female patients with high SAT radiodensity in this study, 35% had a high amount of SAT, meaning that these patients with higher risk of mortality were not captured by measuring SAT quantity, and therefore, SAT radiodensity is a more complete and robust predictor of mortality in these patients. Moreover, this might indicate that morphological rearrangements in SAT occur before the loss of SAT mass.

Fibrosis plays an important role in adipose tissue dysfunction, and excess collagen deposition has been identified as a major contributor to elevated tissue radiodensity in fibrotic cardiovascular adipose tissue.^5^^,^^28^^,^^29^ Furthermore, elevated pressure in the portal vein^4^ and inflammation^3^ were associated with altered adipose tissue radiodensity. Fibrotic and inflamed SAT depots have a detrimental impact on appropriate energy homoeostasis and its endocrine functions. Although dilated interstitial space was noticed on H&E sections in this study, immunohistochemical staining for immune cells and collagen fibre staining for identifying the nature of the extracellular matrix needs to be performed in future studies with a larger number of patients.

High adipose tissue radiodensity might also be related to the tissue-obtaining features of brown adipocyte, which is specialised for dissipating energy as heat.^30^ High SAT radiodensity was described as the mean SAT radiodensity above -83 HU in females and higher than -74 HU in males in this study, which is close to the radiodensity of brown adipose tissue, in the range of -10 to -87 HU.^30^ White adipose tissue browning induced by cancer cachexia and subsequent increase in adipose tissue vascularity and thermogenesis has been recognised as a contributor to adipose atrophy.^31^ Whether increased SAT radiodensity to the range of brown adipose tissue in patients with cirrhosis and poor survival exemplifies white adipose tissue browning requires further investigation. Reversing white adipose tissue browning by β3-adrenergic receptor antagonists or nonsteroidal anti-inflammatory drugs has been proposed in oncology settings^31^; additional research is needed to delineate whether this can be considered as a therapeutic approach to reverse the adipose tissue atrophy in patients with cirrhosis.

Concurrent impacts of age and sex on SAT radiodensity have been investigated in this study. Given 4 combinations of age and sex, high SAT radiodensity was more commonly found in females regardless of age. Body composition assessments in patients with cirrhosis have indicated that fat loss is more common in females, whereas low skeletal muscle is more common in male patients.^32^ Therefore, a higher frequency of high SAT radiodensity in females and low SAT radiodensity in males may suggest that adipose atrophy is more frequent in female patients in response to catabolic stress of the chronic disease, whereas males are primarily using other sources such as muscle in hypercatabolic states. Given a small number of patients with biopsies, the relevant impact of sex on adipose tissue characteristics was not identified in 12 LT recipients.

In this study, mortality risk was evaluated using competitive risk analysis with LT as the competitive event. When a competing risk presents, applying conventional survival analysis could overestimate the risk,^18^ and therefore, a more robust approach was applied in the present study. However, we acknowledge there are limitations in this study caused by its retrospective nature, the long time for enrolling the cases, and the evolution of the radiological and management techniques. In addition, we were not able to evaluate metabolic characteristics such as serum leptin and inflammatory markers in plasma to determine their association with SAT radiodensity. Because of the small number of patients with biopsies, results on the histological features of this pilot observation should be interpreted with caution, and SAT morphological characteristics should be investigated in future studies with a higher number of biopsies. Further, main CT technical factors (thickness of slides or contrast medium administration) need to be consistent between studies to advance the risk stratification in clinical practice using radiologically identified body composition features. However, the impact of contrast medium administration on SAT radiodensity might be negligible as it has been previously reported that there was no difference in SAT radiodensity values between unenhanced and contrast-enhanced CT scans^33^ in patients who underwent assessment for coronary artery disease. In addition, a recent study revealed that IV contrast medium administration can increase SAT mean radiodensity by only 0.8%.^34^ Although validity and generalisability of cut-offs established in this study need to be determined in larger prospective multi-centre studies, to our understanding this study is the largest in North America with almost 800 patients with cirrhosis demonstrating a relationship between SAT radiodensity and survival. Although CT radiodensity might serve as an indirect, non-invasive marker of adipose tissue mass and morphological characteristics, the underlying molecular and structural characterisation of varying CT attenuation still requires further clarification in a larger population of patients with cirrhosis.

In conclusion, high SAT radiodensity is associated with higher mortality in patients with cirrhosis. Subcutaneous adipose tissue morphological rearrangement in patients with high SAT radiodensity may indicate not only diminished lipid stores, but also alterations in cellular and tissue characteristics, evidenced by the reduction in adipocyte size with multilocular cytoplasm. Timely recognition of patients with cirrhosis and high SAT radiodensity may provide us with the opportunity to develop strategies to maintain SAT quantity and quality and prevent further depletion of adipose tissue, which consequently could improve clinical outcomes in cirrhosis.

## Financial support

ME has been awarded the Canadian Institutes of Health Research (CIHR)–Institute of Nutrition, Metabolism and Diabetes (INMD) Fellowship–Hepatology, in partnership with the Canadian Association for the Study of the Liver (CASL) and the Canadian Liver Foundation (CLF; HGY-164788). ME, NMK, JS, DB, VCM, VEB, and AJML have been awarded the Canadian National Transplant Research Program (CNTRP), ATIF Innovation Grant Award 2018. ME, VCM, and AJML have been awarded the University of Alberta Hospital Foundation (UHF) Grant 2018 and the Canadian Liver Foundation (CLF) Grant 2019.

## Authors’ contributions

Conducted data analysis and drafted the first version of the manuscript: ME. Conducted image analysis: ARD. Was involved in data collection and image analysis: ER. Assisted with the study conception, compilation of data, writing of the manuscript, and overseeing of the project: AJML. Contributed to the interpretation of data and revising the manuscript: NMK, JS, DB, KD, VCM, VEB. Commented on the manuscript and approved the final version: all authors.

## Data availability statement

The datasets generated and analysed during the current study are not publicly available but are available from the corresponding author on reasonable request.

## Conflicts of interest

The authors declare no conflicts of interest that pertain to this work.

Please refer to the accompanying ICMJE disclosure forms for further details.

## References

[1] Ebadi M., Bhanji R.A., Tandon P., Mazurak V., Baracos V.E., Montano-Loza A.J. (2020). Review article: prognostic significance of body composition abnormalities in patients with cirrhosis. Aliment Pharmacol Ther.

[2] Carey E.J., Lai J.C., Sonnenday C., Tapper E.B., Tandon P., Duarte-Rojo A. (2019). A North American expert opinion statement on sarcopenia in liver transplantation. Hepatology.

[3] da Cunha A.D.J., Silveira M.N., Takahashi M.E.S., de Souza E.M., Mosci C., Ramos C.D. (2021). Adipose tissue radiodensity: a new prognostic biomarker in people with multiple myeloma. Nutrition.

[4] Ebadi M., Moctezuma-Velazquez C., Meza-Junco J., Baracos V.E., DunichandHoedl A.R., Ghosh S. (2020). Visceral adipose tissue radiodensity is linked to prognosis in hepatocellular carcinoma patients treated with selective internal radiation therapy. Cancers (Basel).

[5] Hanley C., Shields K.J., Matthews K.A., Brooks M.M., Janssen I., Budoff M.J. (2018). Associations of cardiovascular fat radiodensity and vascular calcification in midlife women: the SWAN cardiovascular fat ancillary study. Atherosclerosis.

[6] Gifford A., Walker R.C., Towse T.F., Welch E.B. (2015). Correlations between quantitative fat-water magnetic resonance imaging and computed tomography in human subcutaneous white adipose tissue. J Med Imaging (Bellingham).

[7] Weyer C., Foley J.E., Bogardus C., Tataranni P.A., Pratley R.E. (2000). Enlarged subcutaneous abdominal adipocyte size, but not obesity itself, predicts type II diabetes independent of insulin resistance. Diabetologia.

[8] Baba S., Jacene H.A., Engles J.M., Honda H., Wahl R.L. (2010). CT Hounsfield units of brown adipose tissue increase with activation: preclinical and clinical studies. J Nucl Med.

[9] Din M.U., Raiko J., Saari T., Saunavaara V., Kudomi N., Solin O. (2017). Human brown fat radiodensity indicates underlying tissue composition and systemic metabolic health. J Clin Endocrinol Metab.

[10] Tilves C., Zmuda J.M., Kuipers A.L., Carr J.J., Terry J.G., Wheeler V. (2020). Associations of thigh and abdominal adipose tissue radiodensity with glucose and insulin in nondiabetic African-ancestry men. Obesity (Silver Spring).

[11] Ibrahim M.M. (2010). Subcutaneous and visceral adipose tissue: structural and functional differences. Obes Rev.

[12] Van Harmelen V., Reynisdottir S., Eriksson P., Thörne A., Hoffstedt J., Lönnqvist F. (1998). Leptin secretion from subcutaneous and visceral adipose tissue in women. Diabetes.

[13] Tapper E.B., Zhang P., Garg R., Nault T., Leary K., Krishnamurthy V. (2020). Body composition predicts mortality and decompensation in compensated cirrhosis patients: a prospective cohort study. JHEP Rep.

[14] Shen W., Punyanitya M., Wang Z.M., Gallagher D., St-Onge M.P., Albu Jeanine (2004). Total body skeletal muscle and adipose tissue volumes: estimation from a single abdominal cross-sectional image. J Appl Physiol (1985).

[15] Mitsiopoulos N., Baumgartner R.N., Heymsfield S.B., Lyons W., Gallagher D., Ross R. (1998). Cadaver validation of skeletal muscle measurement by magnetic resonance imaging and computerized tomography. J Appl Physiol (1985).

[16] Miller K.D., Jones E., Yanovski J.A., Shankar R., Feuerstein I., Falloon J. (1998). Visceral abdominal-fat accumulation associated with use of indinavir. Lancet.

[17] Carey E.J., Lai J.C., Wang C.W., Dasarathy S., Lobach I., Montano-Loza A.J. (2017). A multicenter study to define sarcopenia in patients with end-stage liver disease. Liver Transpl.

[18] Austin P.C., Lee D.S., Fine J.P. (2016). Introduction to the analysis of survival data in the presence of competing risks. Circulation.

[19] Montano-Loza A.J. (2014). Skeletal muscle abnormalities and outcomes after liver transplantation. Liver Transpl.

[20] Montano-Loza A.J., Angulo P., Meza-Junco J., Prado C.M.M., Sawyer M.B., Beaumont C. (2016). Sarcopenic obesity and myosteatosis are associated with higher mortality in patients with cirrhosis. J Cachexia Sarcopenia Muscle.

[21] White U.A., Tchoukalova Y.D. (2014). Sex dimorphism and depot differences in adipose tissue function. Biochim Biophys Acta.

[22] Anderson W.D., Soh J.Y., Innis S.E., Dimanche A., Ma L., Langefeld C.D. (2020). Sex differences in human adipose tissue gene expression and genetic regulation involve adipogenesis. Genome Res.

[23] Karastergiou K., Smith S.R., Greenberg A.S., Fried S.K. (2012). Sex differences in human adipose tissues—the biology of pear shape. Biol Sex Differ.

[24] Valencak T.G., Osterrieder A., Schulz T.J. (2017). Sex matters: the effects of biological sex on adipose tissue biology and energy metabolism. Redox Biol.

[25] Murano I., Barbatelli G., Parisani V., Latini C., Muzzonigro G., Castellucci M. (2008). Dead adipocytes, detected as crown-like structures, are prevalent in visceral fat depots of genetically obese mice. J Lipid Res.

[26] Henriques F., Júnior M.L.B. (2020). Adipose tissue remodeling during cancer-associated cachexia: translational features from adipose tissue dysfunction. Immunometabolism.

[27] Ebadi M., Tandon P., Moctezuma-Velazquez C., Ghosh S., Baracos V.E., Mazurak V.C. (2018). Low subcutaneous adiposity associates with higher mortality in female patients with cirrhosis. J Hepatol.

[28] Alvey N.J., Pedley A., Rosenquist K.J., Massaro J.M., O’Donnell C.J., Hoffmann U. (2014). Association of fat density with subclinical atherosclerosis. J Am Heart Assoc.

[29] Shields K.J., El Khoudary S.R., Ahearn J.M., Manzi S. (2017). Association of aortic perivascular adipose tissue density with aortic calcification in women with systemic lupus erythematosus. Atherosclerosis.

[30] Ahmadi N., Hajsadeghi F., Conneely M., Mingos M., Arora R., Budoff M. (2013). Accurate detection of metabolically active “brown” and “white” adipose tissues with computed tomography. Acad Radiol.

[31] Petruzzelli M., Schweiger M., Schreiber R., Campos-Olivas R., Tsoli M., Allen J. (2014). A switch from white to brown fat increases energy expenditure in cancer-associated cachexia. Cell Metab.

[32] Alberino F., Gatta A., Amodio P., Merkel C., Di Pascoli L., Boffo G. (2001). Nutrition and survival in patients with liver cirrhosis. Nutrition.

[33] Monti C.B., Capra D., Malavazos A., Florini G., Parietti C., Schiaffino S. (2021). Subcutaneous, paracardiac, and epicardial fat CT density before/after contrast injection: any correlation with CAD?. J Clin Med.

[34] Troschel A.S., Troschel F.M., Fuchs G., Marquardt J.P., Ackman J.B., Yang K. (2021). Significance of acquisition parameters for adipose tissue segmentation on CT images. AJR Am J Roentgenol.

