# Supplementary material for: Higher subcutaneous adipose tissue radiodensity is associated with increased mortality in patients with cirrhosis
Source: JHEP Rep. 2022 Apr 27;4(7):100495. doi: 10.1016/j.jhepr.2022.100495 (PMC9117882; doi:10.1016/j.jhepr.2022.100495)
Supplement: Multimedia component 1 [file mmc1.pdf]

Higher subcutaneous adipose tissue radiodensity is associated with increased mortality in patients with cirrhosis

Maryam Ebadi, Abha R. Dunichand-Hoedl, Elora Rider, Norman M. Kneteman, James Shapiro, David Bigam, Khaled Dajani, Vera C. Mazurak, Vickie E. Baracos, Aldo J. Montano-Loza

Table of contents

Table S1..2

Table S2..3

Table S1. Clinical Parameters Associated with Mortality in Patients without Sarcopenia

Characteristics	sHR (95% CI)	P-value
**Female Patients**		
ALD cirrhosis	0.47 (0.25-0.91)	0.03
MELD score	1.06 (1.02-1.11)	0.001
Refractory Ascites	2.04 (0.90-4.64)	0.09
Variceal bleeding	3.93 (1.10-14.09)	0.04
Encephalopathy	2.65 (1.23-5.69)	0.01
High SAT Radiodensity (> -83 HU)	1.79 (1.06-3.04)	0.03
**Male Patients**		
MELD score	1.03 (1.00-1.07)	0.049
Refractory Ascites	1.46 (0.84-2.55)	0.18
Encephalopathy	2.33 (1.39-3.89)	0.001
High SAT Radiodensity (> -74 HU)	1.79 (1.02-3.13)	0.04

Abbreviations: AILD, autoimmune liver diseases; CI, confidence interval; MELD, model for end-stage liver disease; SAT, subcutaneous adipose tissue; sHR, sub-distribution hazard ratio
sHRs and P values were estimated using Fine-Gray subdistribution hazard model.

Table S2. Comparison of Adipose Tissue Radiodensity Between Patients with Cirrhosis and Donors

Adipose Tissue Radiodensity	Cirrhosis (n=786)	Donor (n=129)	P-value
SAT radiodensity (HU)	-87±16	-101±8	<0.001
High SAT Radiodensity	203 (26)	2 (2)	<0.001
Visceral adipose tissue radiodensity (HU)	-78±10	-89±8	<0.001

Abbreviations: HU, Hounsfield unit; SAT, Subcutaneous adipose tissue.
High SAT radiodensity was defined as SAT radiodensity >-83 in females and >-74 in males.
